# Successful use of isavuconazole as secondary prophylaxis of cryptococcal meningitis in a person living with HIV and AIDS: a case report

**DOI:** 10.1177/20499361251395903

**Published:** 2026-03-20

**Authors:** Carmela Pinnetti, Alessandro Giacinta, Federico Cecilia, Francesco Baldini, Annalisa Mondi, Saba Gebremeskel Teklè, Susanna Grisetti, Marta Camici, Donatella Vincenti, Stefania Carrara, Carla Fontana, Andrea Antinori

**Affiliations:** Clinical and Research Infectious Diseases Department, National Institute for Infectious Diseases Lazzaro Spallanzani-IRCCS, Rome, Italy; Clinical and Research Infectious Diseases Department, National Institute for Infectious Diseases Lazzaro Spallanzani-IRCCS, Via Portuense 292, Rome 00149, Italy; Clinical and Research Infectious Diseases Department, National Institute for Infectious Diseases Lazzaro Spallanzani-IRCCS, Rome, Italy; Infectious Diseases Clinic, Department of Biomedicine and Prevention, Tor Vergata School of Medicine and Surgery, Rome, Italy; Clinical and Research Infectious Diseases Department, National Institute for Infectious Diseases Lazzaro Spallanzani-IRCCS, Rome, Italy; Clinical and Research Infectious Diseases Department, National Institute for Infectious Diseases Lazzaro Spallanzani-IRCCS, Rome, Italy; Clinical and Research Infectious Diseases Department, National Institute for Infectious Diseases Lazzaro Spallanzani-IRCCS, Rome, Italy; Clinical and Research Infectious Diseases Department, National Institute for Infectious Diseases Lazzaro Spallanzani-IRCCS, Rome, Italy; Clinical and Research Infectious Diseases Department, National Institute for Infectious Diseases Lazzaro Spallanzani-IRCCS, Rome, Italy; Laboratory of Microbiology, National Institute for Infectious Diseases Lazzaro Spallanzani-IRCCS, Rome, Italy; Laboratory of Microbiology, National Institute for Infectious Diseases Lazzaro Spallanzani-IRCCS, Rome, Italy; Laboratory of Microbiology, National Institute for Infectious Diseases Lazzaro Spallanzani-IRCCS, Rome, Italy; Clinical and Research Infectious Diseases Department, National Institute for Infectious Diseases Lazzaro Spallanzani-IRCCS, Rome, Italy

**Keywords:** case report, cryptococcal meningitis, HIV, immunodeficiency, isavuconazole

## Abstract

Cryptococcal meningitis (CM) is a severe opportunistic infection in people living with HIV (PLWH). We report a 54-year-old man with advanced HIV infection who presented with CM due to *Cryptococcus neoformans*. Induction therapy with liposomal amphotericin B (4 mg/kg/day) plus fluconazole (800 mg/day) was prolonged to 10 weeks because flucytosine was initially unavailable; intravenous flucytosine (25 mg/kg q6h) was introduced when accessible. Maintenance fluconazole (800 mg/day) was continued, and antiretroviral therapy (ART) with dolutegravir plus emtricitabine/tenofovir disoproxil was initiated after 8 weeks. One year later, despite virological suppression, he developed neurological deterioration compatible with recurrent CM in the absence of culture confirmation. He underwent re-induction with liposomal amphotericin B plus flucytosine, followed by off-label secondary prophylaxis with oral isavuconazole (200 mg/day). Over 6 months, he maintained HIV-RNA suppression, showed CD4+ T-cell recovery (from 94 to 165 cells/mm^3^), and experienced neurological stabilization without further CM episodes or drug-related toxicity. Isavuconazole’s pharmacokinetic profile, oral availability, and limited antiretroviral drug–drug interactions supported its use as extended secondary prophylaxis in this setting, although limited access in low- and middle-income countries remains a concern. This case highlights isavuconazole as a potential alternative prophylactic strategy when fluconazole is ineffective or not tolerated.

## Introduction

Cryptococcal meningitis (CM) is a severe fungal infection frequently observed in immunocompromised individuals, particularly those living with HIV.^1^ Cryptococcal infections can disseminate to the central nervous system (CNS), causing subacute meningoencephalitis.^2^ Although the incidence of CM has decreased over time in many high-income settings with the introduction of combination antiretroviral therapy (ART), this disease remains associated with high morbidity and mortality among people living with HIV (PLWH), while incidence has remained relatively stable in many low- and middle-income countries (LMICs).^3^

International guidelines for treating CM in PLWH in high-resource settings recommend an induction phase, usually consisting of liposomal amphotericin B (L-AMB) plus flucytosine (5FC) or, if the latter is unavailable, fluconazole, for 2 weeks. This is followed by consolidation therapy with fluconazole for 8 weeks and subsequent secondary prophylaxis with fluconazole, continued until the patient has maintained CD4+ T-cell count greater than 200/mm^3^ for at least 6 months and virologic suppression on ART.^4,5^

Since fluconazole resistance is now spreading in *Cryptococcus* isolates, especially in patients with relapses,^6^ evaluation of the potential use of various triazole drugs, including voriconazole, posaconazole, and isavuconazole (ISAV), for either consolidation or maintenance therapy was attempted, with success rates of approximately 50%.^7–9^

## Methods

We described the management of a case of a PLWH treated at the National Institute for Infectious Diseases in Rome, who experienced clinical deterioration compatible with recurrent CM during fluconazole maintenance, and achieved clinical stabilization following re‑induction and extended maintenance with ISAV.

The study was conducted and reported in accordance with the CARE statement (Supplemental Material).

## Case presentation

In August 2022, a 54-year-old man living with HIV, who was off ART at presentation, was diagnosed with CM caused by *Cryptococcus neoformans* var *neoformans* in our department. The patient had been regularly followed since 2013, but in September 2019, he spontaneously discontinued ART and follow-up visits due to a history of alcohol abuse, substance use (crack), and depression. In July 2022, he presented to our outpatient clinic in poor clinical condition; blood tests documented a CD4+ T-cell count of 20 cells/mm^3^ and an HIV-RNA level of 98,648 copies/mL. The clinical case presentation and treatment outcomes have been previously published.^10^ Focused on CM, at lumbar puncture (LP), the chemical-physical examination of the cerebrospinal fluid (CSF) described an opalescent CSF with unconsumed glucose (128 mg/dL), a mild hyperproteinorrachia (56.6 mg/dL), and a cell count of 239 cells/mm^3^, predominantly lymphomononuclear cells. The culture was positive for *C. neoformans*, susceptible to the main antifungals by EUCAST, with MIC for fluconazole of 4.0, for L-AMB of 0.5, for caspofungin, anidulafungin, and micafungin of 8.0, for voriconazole, posaconazole, and itraconazole of 0.06 and for ISAV of 0.03. The soluble antigen of *C. neoformans* (CrAg) was determined using a latex agglutination test (Pastorex Crypto Plus 61747; Bio-Rad, USA) and was 1:1000 in CSF and 1:1000 in serum, respectively. Opening pressure was not measured; however, CSF outflow was brisk, with closely spaced drops. Identification of *C. neoformans var. neoformans* was performed by matrix-assisted laser desorption/ionization-time of flight (MALDI-TOF).

The patient was initially treated with intravenous L-AMB 4 mg/kg q24h plus fluconazole 800 mg (5FC initially unavailable). When available, after about 8 weeks, intravenous 5FC 25 mg/kg q6h was introduced. Induction was prolonged to 10 weeks; maintenance with fluconazole 800 mg followed. No adverse events attributable to L-AMB were recorded over the prolonged induction. Eight weeks after CM diagnosis, ART with dolutegravir (DTG) and emtricitabine/tenofovir disoproxil (FTC/TDF) was started. Since the change in antifungal therapy, the patient experienced progressive clinical improvement until discharge with fluconazole 400 mg as secondary prophylaxis. At the end of hospitalization, the patient appeared alert and well oriented and was able to walk without assistance; however, he presented with severe visual impairment (confirmed by ophthalmologic evaluation) and severe-to-profound sensorineural hearing loss (for which a cochlear implant had been placed but without functional benefit). He also reported hypoesthesia of the left hand and back-lumbar pain of the lateral muscular fascia.

After a year, in August 2023, he was again admitted to our Department for psychomotor slowing and worsening of mobility with a progressive loss of acquired muscle tone, especially of the right lower limb. ART, which had been changed to bictegravir/emtricitabine/tenofovir alafenamide (BIC/F/TAF) during the outpatient follow-up period (due to mild renal failure), was never suspended, although the CD4+ T cell count was 94 cells/mm^3^ (5.0%) with undetectable HIV-RNA. Adherence to ART and to fluconazole maintenance was assessed during regular follow-up visits; on discharge, the patient was transferred to a rehabilitation facility and subsequently attended scheduled follow-ups.

An LP was again performed: the chemical-physical examination of CSF described a clear liquor with unconsumed glucose (102 mg/dL), a marked hyperproteinorrachia (152.9 mg/dL), and a significant cellularity (225 cells/mm^3^), predominantly lymphomononuclear cells. The culture was negative, while the CrAg and HIV-RNA were 1:200/1:10,000 and 79/<30 cp/mL in the CSF/plasma pairs, respectively.

Alternative AIDS-related CNS diagnoses were investigated: neuroimaging (CT) showed thin, patchy leptomeningeal enhancement, more conspicuous in the right parietal region, judged compatible with the underlying disease. No additional findings were reported. CSF testing for other neurotropic pathogens (Epstein-Barr virus, cytomegalovirus, JC virus, herpes simplex virus-1/2, and varicella-zoster virus, *M. tuberculosis* complex, and nontuberculous mycobacteria) was negative.

**Figure 1.**
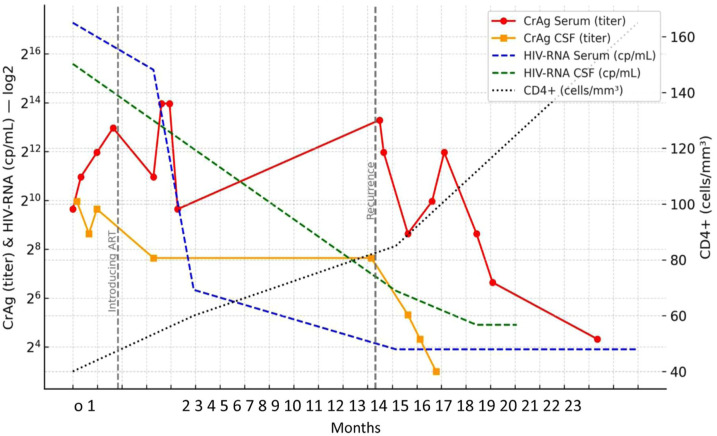
Timeline of clinical presentation, cryptococcal antigen levels in serum and in CSF, HIV-RNA in serum and in CSF, and CD4+ T-cell count. CSF, cerebrospinal fluid.

Managed as clinical recurrence compatible with cryptococcal disease (culture-negative CSF), induction intravenous antifungal treatment with L-AMB and fluconazole (given the temporary unavailability of 5FC) was administered for 10 weeks, followed by maintenance treatment with fluconazole 800 mg once daily. Nonetheless, the neurological picture worsened, with the appearance of weakness in the right upper and lower limbs, inability to coordinate the left hand, psychomotor slowing with progressive loss of motor autonomy, and severe psychomotor agitation. Therefore, an LP was repeated, showing negative culture, but increased CrAg (Figure 1). Opening pressure was not measured. It was therefore decided to start further treatment with L-AMB and 5FC as induction, with subsequent maintenance carried out with ISAV 200 mg once daily off-label. At discharge, the patient underwent rehabilitation with progressive clinical improvement and stability of the neurological picture. Six months later, there had been no relapses of CM, and the patient remained in regular follow-up. Immunologic improvement was documented (CD4+ T-cell count 165 cells/mm^3^, 7.2%), HIV RNA was confirmed at <30 copies/mL, and the serum CrAg titer showed a progressive decline (Figure 1). At that time, functional improvement was observed: the patient was able to ambulate without assistance and to perform basic daily activities. During maintenance with ISAV, adherence was evaluated regularly by structured interview, pill count, and pharmacy refill confirmation. No treatment interruptions were recorded. Safety was monitored with periodic laboratory testing and ECGs; no significant abnormalities or adverse events were observed throughout the months of therapy.

At present, the patient continues regular follow-up and has experienced neurological recovery, although he has not regained either vision or hearing. He has had no further disease episodes and presents a CD4+ T-cell count of 243 cells/mm^3^ (11.4%) with undetectable HIV viral load.

## Discussion and conclusion

This case illustrates the successful use of ISAV as extended secondary prophylaxis following clinical deterioration compatible with cryptococcal disease during fluconazole-based maintenance in a PLWH in a high-resource setting. While conventional induction with L-AMB plus 5FC or fluconazole led to stabilization, sustained clinical control was achieved only after switching maintenance to ISAV, with virological suppression on ART, CD4+ recovery, and improved function at follow-up. Importantly, we do not claim culture-proven relapse or documented fluconazole resistance in this patient.

L-AMB and 5FC are the recommended induction therapy for CNS cryptococcal infections among PLWH in high-resource settings.^11^ Consolidation and maintenance therapy are usually based on fluconazole, although alternative triazoles, including ISAV, have been employed in treatment-failure scenarios.^12^ Itraconazole is less effective than fluconazole for chronic CM and is recommended only as backup treatment.^7,13^ Voriconazole, on the other hand, has a good penetration into the CNS and is recommended as an antifungal agent for invasive infections caused by *Aspergillus* spp., but not by *Cryptococcus* spp., mainly due to toxicity and drug–drug interactions.^14,15^ By contrast, ISAV achieves therapeutic CNS concentrations,^16^ has demonstrated activity against various CNS fungal infections^17^—although it has not been fully investigated in CM cases^9^—, and has been used successfully in induction therapy for CM.^12,18^ Furthermore, its favorable oral bioavailability, reduced toxicity, and limited drug–drug interactions support its potential role in different fungal infections and in long-term prophylaxis in high-risk individuals.^19^ Prior reports described ISAV in CNS fungal infections and, anecdotally, in CM, supporting the biological plausibility of its use as maintenance therapy in difficult cases.^12,18^ In our patient, episodes of clinical deterioration were managed with standard re-induction regimens with L‑AMB plus either 5FC or fluconazole, but only ISAV maintenance achieved sustained clinical stability.

Some limitations must be acknowledged.

First, there was no culture confirmation during clinical worsening, and thus no relapse isolate for susceptibility testing. Previous data showed that people with CM, in whom isolation of *Cryptococcus* species strains documented in vitro resistance to fluconazole, were more likely to experience clinical relapse.^6^ In our case, it was possible to obtain only one sensitivity test on the first CSF culture that was positive for *C. neoformans*. Likely due to the prolonged and uninterrupted antifungal treatment, all subsequent cultures were found to be sterile, and the diagnosis of CM relapse was made exclusively on a clinical basis and by monitoring CrAg both in serum and CSF. In the absence of culture confirmation during clinical worsening, we did not infer fluconazole resistance; nevertheless, we judged the ongoing maintenance therapy ineffective.

Second, intracranial pressure was not recorded at LP, which limits interpretation; however, CSF outflow was brisk, with closely spaced drops, suggesting presumptively elevated intracranial pressure.

Third, access to ISAV remains limited in LMICs.

We also considered CNS immune reconstitution inflammatory syndrome (IRIS) in the differential diagnosis, given the temporal association with ART and immunologic recovery. However, several features make IRIS less likely and favor microbiologic recurrence: first, clinical deterioration occurred well outside the typical 2- to 10-week window after ART initiation that characterizes cryptococcal IRIS^3^; second, deterioration developed despite fluconazole maintenance, alongside rising CrAg titers (supportive of increased antigen burden, though not diagnostic), and improved after re-induction antifungal therapy, a response pattern more compatible with relapse than with an isolated inflammatory IRIS event. Neuroimaging showed leptomeningeal enhancement without alternative AIDS-related CNS diagnoses. We therefore interpret this episode as more consistent with relapse than IRIS, while acknowledging that culture confirmation is required for a definitive diagnosis of relapse.

ISAV was selected over other triazoles for extended secondary prophylaxis because of its favorable pharmacokinetic profile, oral availability, CNS penetration, and lower risk of drug–drug interactions compared with voriconazole or posaconazole.

In addition, considering the need for prolonged therapy times, the choice of antifungal for maintenance therapy must consider the possible risk of long-term toxicity in addition to the possibility of being able to use orally administered drugs. Finally, among people living with HIV, mid- to long-term control of *Cryptococcus* replication in the CNS could result in reduced inflammation and immune activation in the central compartment, often associated with secondary intrathecal HIV replication. In this context, ISAV could be a promising alternative for secondary prophylaxis of CM.

In conclusion, ISAV may represent a reasonable off-label option for extended secondary prophylaxis in selected PLWH with CM when fluconazole is ineffective or poorly tolerated, particularly given its CNS penetration and DDI profile. Prospective data and longer follow-up are warranted to define patient selection, optimal dosing and duration, and comparative effectiveness against other triazoles.

## Supplemental Material

sj-pdf-1-tai-10.1177_20499361251395903 – Supplemental material for Successful use of isavuconazole as secondary prophylaxis of cryptococcal meningitis in a person living with HIV and AIDS: a case report Supplemental material, sj-pdf-1-tai-10.1177_20499361251395903 for Successful use of isavuconazole as secondary prophylaxis of cryptococcal meningitis in a person living with HIV and AIDS: a case report by Carmela Pinnetti, Alessandro Giacinta, Federico Cecilia, Francesco Baldini, Annalisa Mondi, Saba Gebremeskel Teklè, Susanna Grisetti, Marta Camici, Donatella Vincenti, Stefania Carrara, Carla Fontana and Andrea Antinori in Therapeutic Advances in Infectious Disease

## Declarations

**Ethics approval and consent to participate:** Our report did not require ethical board approval as it described the treatment of a single patient, which does not meet the definition of human subjects research. This anonymized case was documented in the context of routine care, and the information presented was anonymized in accordance with the Declaration of Helsinki. The reporting of this study conforms to the Case Report (CARE) guidelines.

**Consent for publication:** The patient reported in this case had preserved decisional capacity at the time of consent despite sensory impairments and provided written informed consent for publication of anonymized clinical information.

**Availability of data and materials:** All data are available as part of this article.
